# Recent advances in understanding neutrophils

**DOI:** 10.12688/f1000research.9691.1

**Published:** 2016-12-23

**Authors:** Justin F. Deniset, Paul Kubes

**Affiliations:** 1 Department of Physiology and Pharmacology, University of Calgary, Calgary, AB, Canada; 2 Calvin, Phoebe, and Joan Snyder Institute for Chronic Diseases, University of Calgary, Calgary, AB, Canada; 3 Department of Microbiology and Infectious Diseases, University of Calgary, Calgary, AB, Canada

**Keywords:** neutrophils, heterogeneity, immunomodulation

## Abstract

Neutrophils have long been regarded as key effectors of the innate immune response during acute inflammation. Recent evidence has revealed a greater functional diversity for these cells than previously appreciated, expanding roles for neutrophils in adaptive immunity and chronic pathologies. In this review, we summarize some of the evolving paradigms in the neutrophil field and highlight key advances that have contributed to our understanding of neutrophil behavior and function *in vivo*. We examine the concept of neutrophil subsets and polarization, we discuss novel immunomodulatory roles for neutrophils in shaping the immune response, and, finally, we identify technical advances that will further enhance our ability to track the function and fate of neutrophils.

## Introduction

Neutrophils are the predominant leukocyte population in human blood and are known as key first responders to sites of injury and infection. Their protection against invading pathogens has been well described with their ability to phagocytose combined with their production and release of reactive oxygen species, proteases, and extracellular traps ^1^. Their role in aiding adaptive immunity is also expanding. Growing research in the field has highlighted roles for neutrophils in numerous inflammatory conditions including sterile injury ^2, 3^, cancer ^4^, atherosclerosis ^5^, and autoimmunity ^6, 7^. The concurrent improvement in the tools and techniques used to evaluate these cells within the *in vivo* setting has further expanded our view of their functions and has challenged existing dogmas within the field. This review aims to highlight some of these emerging concepts and technical advances that have enhanced our understanding of neutrophil function.

## Breaking down neutrophil paradigms

Neutrophils are among the first cells to be recruited to an inflammatory site, where they help to neutralize harmful stimuli. Original studies in the rabbit and rat mesentery and the mouse cremaster muscle identified the key events in neutrophil recruitment in post-capillary venules that included endothelial selectin-dependent tethering and rolling that included rapid expression of P-selectin mediating early (within minutes) rolling and protein-synthesis-dependent E-selectin expression mediating amplification of the rolling (within hours) ^8, 9^. Chemokines such as CXCL8, CXCL1, CXCL2, and CXCL3 as ligands for the CXCR2 receptor activate the neutrophils to adhere via CD11a/CD18 and then crawl to junctions via CD11b/CD18, where they emigrate via CD31 JAMs and CD99 ^8, 9^. While this was initially thought to be the general scheme for neutrophil recruitment, it appears that this recruitment cascade may predominate in places like the muscle, skin, brain, and perhaps heart (although less is known about the latter). In direct contrast, selectins appear to be much less important in places like the liver and lung ^10, 11^ (P. Kubes and B. Yipp, unpublished observations). In addition, there is mounting evidence that while selectins may be important for neutrophil recruitment in the kidney ^12– 14^, platelets rather than the endothelium are the source of these adhesion molecules and the glomeruli capillaries are the site of adhesion ^15^. Also, certain stimuli in the liver induce a CD44-dependent neutrophil adhesion while other stimuli make use of integrins ^2, 16^. In the lung, there is much evidence that neither integrins nor CD44 are important for neutrophil adhesion in pulmonary capillaries ^11, 17^ (P. Kubes and B. Yipp, unpublished observations). Clearly, the old textbook universal paradigm for leukocyte recruitment that invokes selectins and integrins is slowly changing.

Another important issue that has recently been debated is the lifespan of neutrophils. While these cells in humans were thought to be short lived (8–12 hours) for many years, recent work has suggested that these cells may live for up to 5 days in the circulation ^18^. Some have challenged this latest concept, with a main criticism stating that the labeling technique utilized likely also labeled bone marrow neutrophils ^19, 20^. A shorter lifespan of 8–12 hours has also been noted in mice ^21, 22^. Casanova-Acebes *et al*. showed that neutrophils undergo aging in the circulation by increasing their expression of CXCR4 and reducing their expression of CD62L prior to their return to the bone marrow following a circadian pattern ^22^. Using adoptive transfer of newly emigrated neutrophils (CXCR4 ^lo^ CD62L ^hi^), the authors noted a transition to an aged phenotype and subsequent removal from the circulation within 8 hours ^22^. Work from Zhang *et al*. has further demonstrated that the microbiome is involved in driving this aged phenotype, as they noted reductions in circulating aged neutrophils in germ-free and antibiotic-treated animals ^23^. In addition to their circulation in the bloodstream, neutrophils can also enter some compartments such as the spleen and lung, forming marginated pools under steady-state conditions ^24– 26^. These marginated neutrophils are noted to play homeostatic functions and have the ability to mobilize back into the circulation ^24, 26, 27^. However, their residence time within these peripheral sites is still an area of uncertainty. This can be further confounded by the role of peripheral tissues in neutrophil homeostasis. Uptake of apoptotic neutrophils in peripheral sites (e.g. the intestine, spleen, and lung) by macrophages and dendritic cells provides negative feedback signals for the IL-23/IL-17/G-CSF axis that regulates granulopoiesis ^28, 29^. The lifespan of neutrophils can be prolonged upon activation, which is thought to ensure the presence of primed neutrophils at the site of inflammation ^1, 30^. Recent work, primarily in zebrafish embryos, has noted that neutrophils could enter sites of sterile injury and then return back to the circulation (termed reverse transmigration) ^31– 33^. Although this has not been shown in mammalian cells, one group has shown that neutrophils could at least extend a pseudopod or even their whole body out of the vasculature before returning back into the circulation ^34^. As such, reverse transmigration may represent a key mechanism to extend neutrophil lifespan in the context of inflammation.

Finally, until about 10 years ago, neutrophils were thought to catch and kill bacteria via opsonization, phagocytosis, and oxidant- and protease-dependent killing. In 2004, Zychlinsky and colleagues first demonstrated that neutrophils could, in a last-gasp effort to kill bacteria, release all of their cytotoxic molecules on a DNA backbone, forming what are now known as neutrophil extracellular traps (NETs) ^35^. The adhesive nature of DNA combined with proteases on their surface helps NETs to catch and ultimately kill or at least immobilize various bacteria. This explains why DNAse is a virulence factor in numerous bacterial strains, as it helps their escape from NETs, and it also explains why histones are so potently anti-microbial.

A subsequent study, however, showed that NET production could lead to injury and raised the possibility that this could be a mechanism by which neutrophils contribute to inappropriate inflammatory situations ^36^. In that study, a very clear collaboration of platelets binding to neutrophils was observed before NETs were produced, and the authors suggested that platelets function as a barometer for neutrophils to make NETs when bacteria and their products exceed a platelet-tolerable level. Since that publication, a very significant number of studies have begun to further show that platelets collaborate with neutrophils on many different aspects of their function including chemotaxis and recruitment in addition to bacterial killing ^37– 42^.

## Neutrophil heterogeneity and plasticity

The concept of neutrophil heterogeneity has emerged with accumulating evidence of neutrophil populations with distinct functions under both homeostatic and pathological conditions. Many strategies have been used to identify neutrophil populations, including distinct cell surface markers, cell maturity, function, and residency. Subsets of human circulating neutrophils have been identified under steady-state conditions based on their expression of CD177 or OLFM4 and linked them with autoimmunity ^43, 44^. It should be noted that there is considerable variability in CD177 expression within the population and it is not expressed by all individuals ^45^. Pro-angiogenic neutrophils that are CD49d ^+^VEGR1 ^hi^CXCR4 ^hi^ are also present in low quantities in both mouse and human blood and are recruited in response to VEGF-A ^46, 47^. Aged neutrophils and neutrophils that have undergone reverse transmigration also display changes in their cell surface markers ^1, 48^. In addition to these examples, the prevalence of novel neutrophil populations has recently been identified during infection ^49– 52^, autoimmunity ^53^, cancer ^54– 56^, cardiovascular disease ^57, 58^, and pregnancy ^59^. Despite our advancement in the identification of different neutrophil populations, it is still not understood whether these examples are distinct neutrophil subsets that derive from separate lineages or simply represent activation or polarization states of a common plastic neutrophil precursor.

The cancer field in particular has struggled with these questions. The involvement of neutrophils has been linked to many steps of tumor progression, including initiation, growth, and metastasis for which the numerous mechanisms have been highlighted in recent reviews ^4, 60, 61^. Contradictory roles for neutrophils in this disease setting have been established, with a large proportion of studies identifying pro-tumoral functions ^62– 67^ while others demonstrate anti-tumor properties ^68– 72^. Fridlender *et al*. first introduced the concept of N1/N2 polarization states in an effort to reconcile some of the opposing functions for tumor-associated neutrophils (TANs) ^54^. Based on similarities in function to tumor-associated macrophages (TAMs), they termed these populations N1 and N2 to describe anti-tumor and pro-tumor neutrophil populations, respectively. They provided the first evidence *in vivo* to demonstrate that TGF-β produced by the local tumor microenvironment could polarize a mature neutrophil to adopt a pro-tumor N2 phenotype ^54^. Since then, angiotensin-II, type I IFNs, and the proto-oncogene *MET* have also been shown to promote N2 or N1 polarization states ^68, 73, 74^.

A similar neutrophil phenotypic duplicity has been identified in the blood of tumor-bearing mice and cancer patients based on separation by density gradient ^55, 75^. In these samples, elevated levels of both immature and mature neutrophil populations were found in the low-density fraction, collectively termed low-density neutrophils (LDNs), in addition to the previously named tumor-entrained neutrophils (TENs) in the high-density layer, termed high-density neutrophils (HDNs). The HDN and LDN populations displayed anti-tumor and pro-tumor functions, respectively ^55^. Using adoptive transfer approaches, Sagiv *et al*. demonstrated that HDNs could transition to mature LDNs both in tumor-bearing mice and during self-resolving peritonitis ^55^. They also showed that, similar to the polarization of N2 neutrophils in the tumor microenvironment, TGF-β is a key determinant for this conversion in blood ^55^. A subsequent study by Guglietta *et al*. provided additional evidence for this transition, which was driven by NET-induced blood clots in an intestinal cancer model ^75^. Transcriptome evaluation in this study supported the idea that LDNs represent a transitory phenotype between HDNs and N2 TANs ^75^. The similarities between the N1/N2 polarized neutrophils and circulating mature HDNs/LDNs have prompted a new nomenclature of N _C1_ and N _C2 _for these latter populations, respectively ^55^. Although this nomenclature may help to distinguish between overall pro- and anti-tumor functions, the lack of specific markers for these polarized states makes it difficult to evaluate distinct populations and the relationships that may exist between them.

The evaluation of neutrophils in cancer is further complicated by the inclusion of myeloid-derived suppressor cells (MDSCs), which are characterized as a heterogeneous population of CD11b ^+^Gr-1 ^+^ myeloid cells at different stages of differentiation with immunosuppressive functions ^76, 77^. Subsequent analysis has described two populations, granulocytic MDSCs (G-MDSCs) and monocytic MDSCs (M-MDSCs), based on their expression of Ly6G and Ly6C ^76^. The overlapping markers between G-MDSCs and TANs have made it difficult to distinguish between these populations; however, recent transcriptome analysis has noted differences between G-MDSCs and N2 TANs, suggesting that they are in fact distinct populations ^78^. It is still unclear whether G-MDSCs represent a separate lineage of cells or a polarized immature neutrophil. Sagiv *et al*. have proposed that G-MDSCs represent the immature LDN population ^55^. Although some have postulated that G-MDSCs could potentially give rise to N2 or N _C2_, there is little evidence to support this concept. Furthermore, an immature phenotype in the cancer setting may also not be limited to G-MDSCs and their immunosuppressive function. Singhal *et al*. recently reported that immature neutrophils contribute to a hybrid antigen-presenting neutrophil found in early lesions in humans ^56^. They demonstrated that only immature neutrophil populations and not mature neutrophils could differentiate into this anti-tumor phenotype in response to low levels of GM-CSF and IFN-γ, as encountered in the tumor microenvironment ^56^. Further, by simply increasing the levels of IFN-γ, they also noted the generation of hybrid neutrophils with immunosuppressive functions ^56^. A similar hybrid phenotype was also noted in a separate study when immature neutrophils were exposed to only GM-CSF ^79^. This highlights the innate plasticity of immature neutrophils with their ability to integrate multiple signals to drive their phenotype. Importantly, it also supports the concept that polarization does not have to be unidirectional and that it can lead to the acquisition of multiple functions simultaneously.

These polarization schemes have also been adopted in other inflammatory pathologies. Cuartero *et al*. demonstrated the identification of an N2 subset in the injured brain following stroke ^57^. In this model, PPAR-gamma agonist-mediated protection was dependent on neutrophils and coincided with an increase in the proportion of N2 neutrophils ^57^. Recently, a temporal polarization of N1 to N2 neutrophils in the heart following myocardial infarction was also noted ^58^. These studies support the expanded view that N2 can also contribute to resolution and tissue repair. LDN fractions have also been noted in patients with systemic lupus erythematosus; however, in this context, they displayed a pro-inflammatory phenotype ^53^. These examples highlight that the current N1/N2 and HDN/LDN (N _C1_/N _C2_, G-MDSCs) nomenclature is likely an oversimplification and does not capture the spectrum of phenotypes that are likely present both at the inflammatory site and in the circulation under different pathological conditions. Similar issues with the M1/M2 model in the macrophage field have prompted reevaluation and the conceptualization of new nomenclature and polarization models ^80, 81^. These may serve as appropriate templates for defining polarization in neutrophils, which will be important given the constant identification of neutrophil populations with novel functions.

## Immunomodulation by neutrophils

It is well established that neutrophils are rapidly recruited to sites of inflammation where they are able to eliminate harmful stimuli through direct mechanisms ^1^. Recent findings have identified new strategies by which neutrophils also contribute to the progression of the immune responses by modulating the function of other components of the innate and adaptive immune systems.

Neutrophils drive the amplification of the inflammatory response through the release of chemokines and granule proteins, which contribute to the recruitment of additional neutrophils, monocytes, dendritic cells, and lymphocytes. Indeed, neutrophils, through the release of CCL3, recruit dendritic cells while neutrophil-derived CRAMP induces monocyte recruitment ^82, 83^. However, it is worth mentioning that monocyte recruitment can occur independently of neutrophil-derived cues, as depletion of neutrophils in a liver sterile injury model did not influence the accumulation of monocytes at the inflammatory site ^84^. New evidence has extended this guidance function for neutrophils to the recruitment of lymphocytes. Lim *et al*. showed that in response to influenza infection in mice, neutrophils that migrate to the infection site deposit membranous trails in the interstitial tissue containing the chemokine CXCL12 to serve as a chemokine map for migrating CD8 ^+^ T cells ^85^. The blockade of this mechanism resulted in worse outcomes to the infection ^85^. A recent study identifies a similar guidance role for neutrophils in iNKT cell recruitment out of the lung ^86^. In response to *Streptococcus pneumoniae* infection, iNKT migration out of the vasculature and subsequent activation in the interstitial space was dependent on prior transmigration of neutrophils. This mechanism was mediated by neutrophil release of CCL17, and blocking this signal disrupted iNKT localization and activation and increased susceptibility to *S. pneumoniae* infection ^86^. In fact, simply using the neutrophil chemokine CXCL1 to elicit neutrophil migration out of the pulmonary vasculature also induced iNKT cell emigration. Another intravital imaging study in skin elegantly delineates how neutrophils have the capacity to modulate the behavior of other neutrophils locally. Using a sterile injury model, Lammermann *et al*. demonstrated that select neutrophils localize to the sterile injury site, undergo cell death, and release leukotriene B4 (LTB4) to initiate swarming of neutrophils in the area to the site of injury ^3^. As such, these leading neutrophils serve as a beacon of sorts to locally amplify neutrophil recruitment to the injury more precisely. This mechanism is also observed during infection ^3^. This new evidence highlights the important role that neutrophils play in shaping the type of immune response locally at the site of inflammation.

In addition to this guiding mechanism, neutrophils can also play an immunomodulatory role by priming or activating immune cells to promote an effector function. Different neutrophil populations have the ability to promote or suppress T cell activation and subsequent proliferation *in vitro* ^52, 55^. *In vivo* studies further note the capacity of neutrophils to transport antigen to the lymph node and bone marrow ^87, 88^ and promote direct or indirect cross-presentation of antigen to T cells ^88, 89^. Recently, Hampton *et al*. combined the use of a photoconvertible mouse system with intravital microscopy to accurately track the fate of neutrophils following *Staphylococcus aureus* skin infection ^90^. Using this platform, they clearly demonstrated that neutrophils recruited in response to skin infection can than egress to the draining lymph and drive both CD4 and CD8 T cell responses ^90^. Alternatively, recent evidence has also demonstrated a T-cell-suppressive function for neutrophils, particularly in the context of cancer. Using a lung cancer model, Coffelt *et al*. demonstrated that neutrophils recruited to the pre-metastatic niche inhibit CD8 ^+^ T cell activity via an iNOS-dependent mechanism to promote tumor metastasis ^62^.

Neutrophils are equally important determinants of humoral responses. A subset of neutrophils, termed B-helper neutrophils, found within the perifollicular zone of human spleens have the capacity to induce T-independent antibody production by marginal zone B (MZB) cells *in vitro,* possibly via the production of B-cell-stimulating factors (e.g. BAFF, APRIL, and IL-21) ^26^. This observation has been recently extended *in vivo* to mice, where Chorny *et al*. recently demonstrated that pentraxin 3 (PTX3) produced by B-helper neutrophils contributes to this MZB cell activation ^27^. This function is likely due to spleen-specific polarization of neutrophils, as circulating neutrophils from patients could not initiate the same response ^26^. In fact, innate lymphoid cells and endothelial cells within the spleen are believed to at least partly drive the generation of this B-helper neutrophil subset via GM-CSF- and IL-10-mediated mechanisms ^26, 91^. Furthermore, neutropenic patients also display reduced levels of antibodies to T-independent antigen ^26^. Conversely, Kamenyeva *et al*. demonstrated that neutrophils recruited to the lymph following immunization or infection of the skin suppress antibody production by follicular B cells ^92^. They showed by intravital microscopy that, upon entry into the lymph node from the circulation, neutrophils form long-term interactions with both B cells and plasma cells and block their ability to make antibodies through the secretion of TGF-β ^92^.

Neutrophil-mediated priming can also induce effector maturation of macrophages *in vivo*. Using a parasitic infection model, Chen *et al*. showed that neutrophils within infected animals entrained long-term alternative macrophage polarization that was crucial to the clearance of the parasite ^49^. They proposed that neutrophils mediate this regulation through the release of IL-13 ^49^. More recently, Warnatsch *et al*. identified a novel role for NETs in priming macrophage inflammasome activity, which when given in combination with cholesterol crystals led to the production of IL-1β ^93^. They proposed that the release of this cytokine led to the recruitment of Th17 cells and this in turn promoted further recruitment of neutrophils, perpetuating a chronic inflammatory cycle within the atherosclerotic lesion. Importantly, the use of NET-deficient animals or their breakdown by DNase treatment resulted in reduced atherosclerotic plaque development and inflammatory status within the vascular wall ^93^. In contrast, NET production in gout patients resulted in the sequestration of cytokines and the resolution of inflammation in this acute inflammatory condition. These newly described mechanisms could have important applications in other Th2 (e.g. allergies) and chronic inflammatory (e.g. rheumatoid arthritis) settings, respectively.

## Technical advances and future directions

Novel technologies have been created or applied in the last few years to enhance our ability to track and characterize neutrophils in the *in vivo* setting. Until recently, non-specific reporter mice including the LyzM mouse that reported on myeloid cells and the GR-1 antibody that reported on myeloid cells at low concentrations and depleted both neutrophils and monocytes at higher concentrations left ambiguous conclusions in the neutrophil field. The development of the neutrophil-specific “Catchup” reporter mouse has been long overdue and an important advancement in the field. To achieve this, Hasenberg *et al*. created a cre-based reporter system that is driven by the Ly6G promoter and paired with the dtTomato reporter ^94^. This provided an upgrade for intravital imaging users over the much-utilized LysM-GFP reporter mouse, as it does not label both monocytes and macrophages. These authors demonstrated that disrupting both Ly6G alleles did not result in any apparent defects in an exhaustive number of different models. This work gives credence to results using the Ly6G antibody as a neutrophil lineage-specific reagent that can selectively deplete this cell type. Just as important, this work points to the possibility of generating conditional neutrophil-specific knockout animals using the Ly6G cre recombinase, complementing the recently described approach using the Mrp8-promoter-driven cre recombinase system for specific deletion of Card9 and MET ^68, 95^.

Photoconversion of neutrophils *in situ*, as demonstrated by Hampton *et al*. ^90^, provides a powerful tool in evaluating the fate of neutrophils at the site of inflammation. This technique has also been employed to study reverse transmigration back into the circulation in zebrafish ^33^. It should be mentioned that these examples use preparations that are easily accessible and/or transparent, thus optimal for light penetration and activation. The challenge will be to apply this approach to organs in mice that lack the same transparent properties. Morton *et al*. demonstrated the effectiveness of an endoscopic approach for the activation and tracking of lymphocytes in the gut ^96^. Furthermore, increasing the precision of this platform with the use of photoconvertible and photoactivatable reporters driven by a neutrophil-specific promoter (e.g. Ly6Gcre) would enhance our ability to track neutrophils by intravital microscopy. Another issue in the neutrophil field is that sterile injury of any kind induces neutrophil recruitment, and so preparations without the need for invasive surgery would certainly benefit the field. The use of a cranial or abdominal chronic window could serve as a solution for longer-term tracking in the brain, liver, spleen, and kidney ^97, 98^. However, the recent observations that peritoneal macrophages invade visceral organs directly from the peritoneum ^99^ could be affected by immobilizing a window on the tissue.

In order to better understand the dynamics of neutrophil heterogeneity, in-depth profiling of these cells both temporally and spatially will be important. The recently developed platforms of mass cytometry, histo-cytometry, and next-generation sequencing (e.g. ChIP-seq, ATAC-seq, and RNA-seq) may prove to be useful in this regard. Mass cytometry, which labels cells with the use of heavy metal conjugated antibodies and subsequently runs them through a mass spectrometer, removes the requirement for compensation and as such can accommodate larger antibody panel sizes than conventional flow cytometry systems. Becher *et al*. have recently used a 38-marker panel to characterize the mouse myeloid compartment and demonstrated the ability to identify five different neutrophil populations ^100^. However, whether this was simply a reflection of different ages of neutrophils or different environmental modulations of the same cell remains to be determined. Histo-cytometry combines immunohistochemistry with cytometry software in order to spatially display different cell subsets within a given tissue. This technology has recently been used to distinguish the different dendritic cell populations in the lymph node ^101^. Next-generation sequencing uses high-throughput platforms that can provide information regarding cell-specific transcriptome and gene regulation profiles. Combinations of these platforms, including single-cell sequencing approaches, have recently been employed to characterize myeloid progenitor and myeloid populations including neutrophils, defining signatures for different subsets ^62, 102– 104^. Collectively, these techniques could provide additional context to functional differences between neutrophil subsets within a particular inflammatory setting, such as N1 and N2 neutrophils within the tumor microenvironment.

Neutrophils are now known to play important roles in many pathologies, including cancer, cardiovascular disease, and autoimmunity. This knowledge, combined with the emergence of novel immunomodulatory functions and phenotypes for neutrophils, has helped to re-invigorate interest in the field. Targeting the mechanisms that regulate these functions has proven to be a promising therapeutic approach in numerous experimental settings. The key challenge moving forward is integrating these concepts within the human context.
